# Impact of HIV-1 Vpu-mediated downregulation of CD48 on NK-cell-mediated antibody-dependent cellular cytotoxicity

**DOI:** 10.1128/mbio.00789-23

**Published:** 2023-07-05

**Authors:** Lorie Marchitto, Mehdi Benlarbi, Jérémie Prévost, Annemarie Laumaea, Jade Descôteaux-Dinelle, Halima Medjahed, Catherine Bourassa, Gabrielle Gendron-Lepage, Frank Kirchhoff, Daniel Sauter, Beatrice H. Hahn, Andrés Finzi, Jonathan Richard

**Affiliations:** 1 Centre de Recherche du CHUM, Montreal, Quebec, Canada; 2 Département de Microbiologie, Infectiologie et Immunologie, Université de Montréal, Montreal, Quebec, Canada; 3 Institute of Molecular Virology, Ulm University Medical Center, Ulm, Germany; 4 Institute for Medical Virology and Epidemiology of Viral Diseases, University Hospital Tübingen, Tübingen, Germany; 5 Department of Medicine, Perelman School of Medicine, University of Pennsylvania, Philadelphia, Pennsylvania, USA; 6 Department of Microbiology, Perelman School of Medicine, University of Pennsylvania, Philadelphia, Pennsylvania, USA; McMaster University, Hamilton, Ontario, Canada

**Keywords:** HIV-1, NK cell, Vpu, Nef, CD48, NTB-A, SLAM, CD4, BST-2, ADCC

## Abstract

HIV-1 evades antibody-dependent cellular cytotoxicity (ADCC) responses not only by controlling Env conformation and quantity at the cell surface but also by altering NK cell activation via the downmodulation of several ligands of activating and co-activating NK cell receptors. The signaling lymphocyte activation molecule (SLAM) family of receptors, which includes NTB-A and 2B4, act as co-activating receptors to sustain NK cell activation and cytotoxic responses. These receptors cooperate with CD16 (FcγRIII) and other activating receptors to trigger NK cell effector functions. In that context, Vpu-mediated downregulation of NTB-A on HIV-1-infected CD4 T cells was shown to prevent NK cell degranulation via an homophilic interaction, thus contributing to ADCC evasion. However, less is known on the capacity of HIV-1 to evade 2B4-mediated NK cell activation and ADCC. Here, we show that HIV-1 downregulates the ligand of 2B4, CD48, from the surface of infected cells in a Vpu-dependent manner. This activity is conserved among Vpu proteins from the HIV-1/SIVcpz lineage and depends on conserved residues located in its transmembrane domain and dual phosphoserine motif. We show that NTB-A and 2B4 stimulate CD16-mediated NK cell degranulation and contribute to ADCC responses directed to HIV-1-infected cells to the same extent. Our results suggest that HIV-1 has evolved to downmodulate the ligands of both SLAM receptors to evade ADCC.

**IMPORTANCE:**

Antibody-dependent cellular cytotoxicity (ADCC) can contribute to the elimination of HIV-1-infected cells and HIV-1 reservoirs. An in-depth understanding of the mechanisms used by HIV-1 to evade ADCC might help develop novel approaches to reduce the viral reservoirs. Members of the signaling lymphocyte activation molecule (SLAM) family of receptors, such as NTB-A and 2B4, play a key role in stimulating NK cell effector functions, including ADCC. Here, we show that Vpu downmodulates CD48, the ligand of 2B4, and this contributes to protect HIV-1-infected cells from ADCC. Our results highlight the importance of the virus to prevent the triggering of the SLAM receptors to evade ADCC.

## INTRODUCTION

Antibody-dependent cellular cytotoxicity (ADCC) represents a major mechanism used by the immune system to target and eliminate virally infected cells (1 - 10). This adaptive host immune response is largely mediated by natural killer (NK) cells and results in the lysis of cells exposing cell-surface antigens bound by specific antibodies (Abs) (11). Cross-linking of the NK cell CD16 (FcγRIII) receptor by bound Abs triggers NK cell effector functions leading to the release of cytotoxic molecules and the cytolysis of the target cells (11). NK cells were shown to contribute to the elimination of HIV-1-infected cells by ADCC both *in vitro* (12 - 18) and *in vivo* (19 - 22), thus highlighting the potential of harnessing this immune response for cure strategies.

HIV-1 evolved different mechanisms to escape ADCC responses, including the downregulation of CD4, BST-2, and several stress ligands. Its accessory proteins Nef and Vpu play a central role in these activities by hijacking protein trafficking machineries. Env is the only target for ADCC-mediating antibodies (Abs), which are present in the plasma of people living with HIV. These commonly elicited Abs preferentially recognize CD4-induced (CD4i) Env epitopes (13, 23), which are occluded in the native closed trimer (12, 24 - 27). However, they are exposed in the CD4-bound “open” Env conformation, which can be triggered by interaction with cell surface CD4 that is incompletely downregulated (12, 13, 28, 29). Thus, Nef and Vpu protect infected cells from ADCC responses by preventing the sampling of Env “open” conformation. Downregulation of cell-surface CD4 by both viral proteins prevents exposure of CD4i Env epitopes, while Vpu-mediated antagonism of the restriction factor BST-2 prevents cell-surface Env accumulation (12 - 15).

Cytolytic NK cell activities do not solely rely on CD16 stimulation but are also tightly regulated by a complex array of activating, co-activating, and inhibitory receptors targeting various molecules at the surface of target cells. The balance between the activating and inhibitory signals delivered by these receptors controls NK cell cytotoxic responses directed against target cells [reviewed in references (30 - 32)]. NK cell activating receptors, such as DNAM-1 (CD226) and NKG2D (CD314), can promote NK cell activation and degranulation (33, 34). HIV-1 Nef and Vpu interfere with NK cell activation by downregulating several ligands of DNAM-1 and NKG2D from the surface of infected cells (35 - 37). As both activating receptors act as co-receptors of CD16 to mediate NK cell-mediated ADCC responses (32, 37, 38), this contributes to reduce the susceptibility of HIV-1-infected cells to both direct and Ab-dependent NK cell responses (35 - 39 - 39).

It has been well established that activating receptors cannot trigger NK cell effector functions on their own but depend on the co-engagement of NK cell co-activating receptors (32). Members of the signaling lymphocyte activation molecule (SLAM) family of receptors, which include NTB-A (CD352/SLAMF6) and 2B4 (CD244/SLAMF4), act as co-activating receptors on NK cells, sustaining their activation and cytotoxic responses (40 - 43). Both receptors were found to synergize with several activating receptors (44 - 46), as well as CD16, to stimulate NK cell effector functions (37, 45). NTB-A and 2B4 play a critical role in the NK cell response against several viruses, including Epstein-Barr virus (EBV), influenza virus, cytomegalovirus (CMV), and HIV-1 (40, 47 - 50). Notably, in patients with the X-linked lymphoproliferative disease, the inability to control EBV infection was proposed to be the consequence of major dysfunctions in both NTB-A and 2B4 (40, 49, 50). NTB-A is expressed on all human NK, T, and B cells (40). Like other SLAMs, NTB-A is homophilic, therefore acting as its own ligand. NTB-A present on NK cells can stimulate NK cell effector function through an homophilic interaction with NTB-A present on target cells (51). In agreement with this, the downmodulation of NTB-A from the surface of HIV-1-infected CD4 T cells by Vpu was shown to prevent NK cell degranulation and reduce the susceptibility of infected cells to NK cell responses, including ADCC (37, 46). While Vpu induces the degradation of BST-2 and CD4, it does not degrade NTB-A but rather affects its glycosylation and anterograde transport (46, 52). Vpu-mediated NTB-A downregulation requires an interaction between the transmembrane domain (TMD) of both proteins (46). Like NTB-A, 2B4 is expressed by human NK cells and can trigger NK cell degranulation in cooperation with NKG2D, DNAM-1, or CD16 (45). In contrast to NTB-A and the other SLAMs, 2B4 does not act as its own ligands but interacts with CD48 (SLAMF2), a GPI-anchored protein broadly expressed on leukocytes (53, 54). Here, we examined whether HIV-1 infection also impairs CD48-2B4 interaction. We show that HIV-1 downregulates cell-surface CD48 in a Vpu-dependent manner, thereby reducing the susceptibility of HIV-1-infected CD4 T cells to ADCC responses. Like NTB-A, we demonstrate that the downmodulation of CD48 by Vpu is a conserved activity among the *vpu*-encoding precursors of the HIV-1/SIVcpz lineage. Our results suggest that HIV-1/SIVcpz have evolved to prevent the triggering of both SLAM to evade ADCC.

## RESULTS

### HIV-1 downregulates CD48 from the cell surface

**Fig 1:**
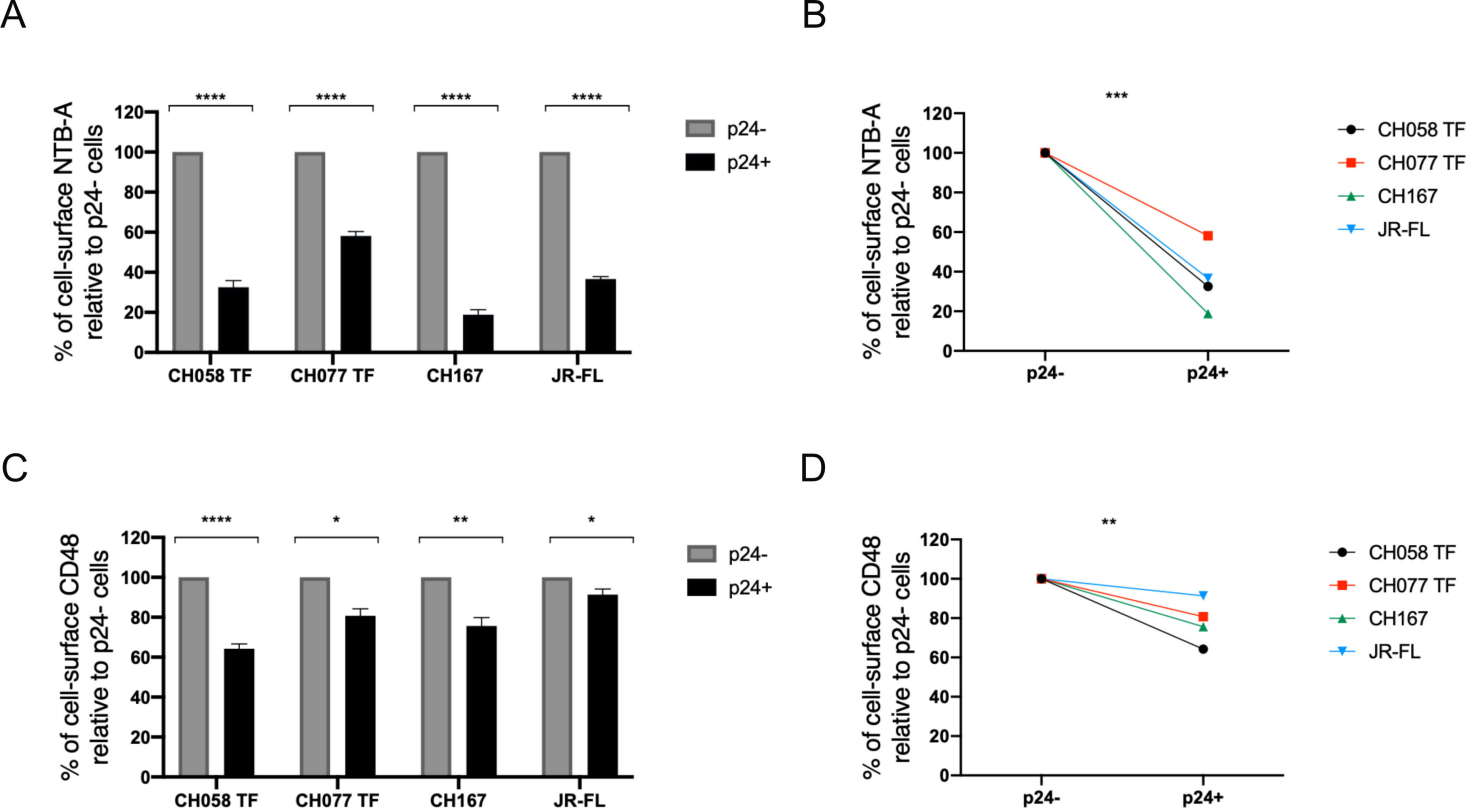
HIV-1 downregulation of NTB-A and CD48. Primary CD4^+^ T cells were infected with indicated IMCs. At 48 h post infection, cells were stained with anti-NTB-A and anti-CD48 Abs, followed with the appropriate secondary Abs. (**A and C**) The graphs shown represent the percentage of median fluorescence intensities (MFI) of (**A**) NTB-A or (**C**) CD48 detected on the surface of p24^+^ cells (black bars) relative to the bystander p24^−^ cells (gray bars) for at least four independent experiments. (**B and D**) Graph representing the mean percentage of surface expression for (**B**) NTB-A and (**D**) CD48 for each tested virus. Error bars indicate means ± standard errors of the means (SEM). Statistical significance was tested using unpaired *t*-tests or Mann-Whitney *U* tests based on statistical normality (*P < 0.05; **P < 0.01; ***P < 0.001; ****P < 0.0001; ns, non-significant).

The ability of HIV-1 to downregulate NTB-A from the surface of primary CD4^+^ T cells is well established (37, 46). However, less is known about the ability of HIV-1 to modulate CD48, the ligand of 2B4. We, therefore, examined the expression of CD48 on the surface of HIV-1-infected primary CD4 T cells. Activated primary CD4^+^ T cells isolated from healthy HIV-1-negative individuals were infected with a panel of wild-type (WT) full-length infectious molecular clones (IMCs), including transmitted founder (TF) viruses. The surface levels of NTB-A (as a control) and CD48 were monitored 48 h post infection by flow cytometry. All tested viruses downregulated NTB-A and CD48 from the surface of infected (p24^+^) cells relative to uninfected (p24^−^) cells (Fig. 1A and C). Despite variation among the different IMCs tested, both ligands were significantly downregulated by all viruses (Fig. 1B and D).

### HIV-1 Vpu is necessary and sufficient for CD48 downregulation

**Fig 2:**
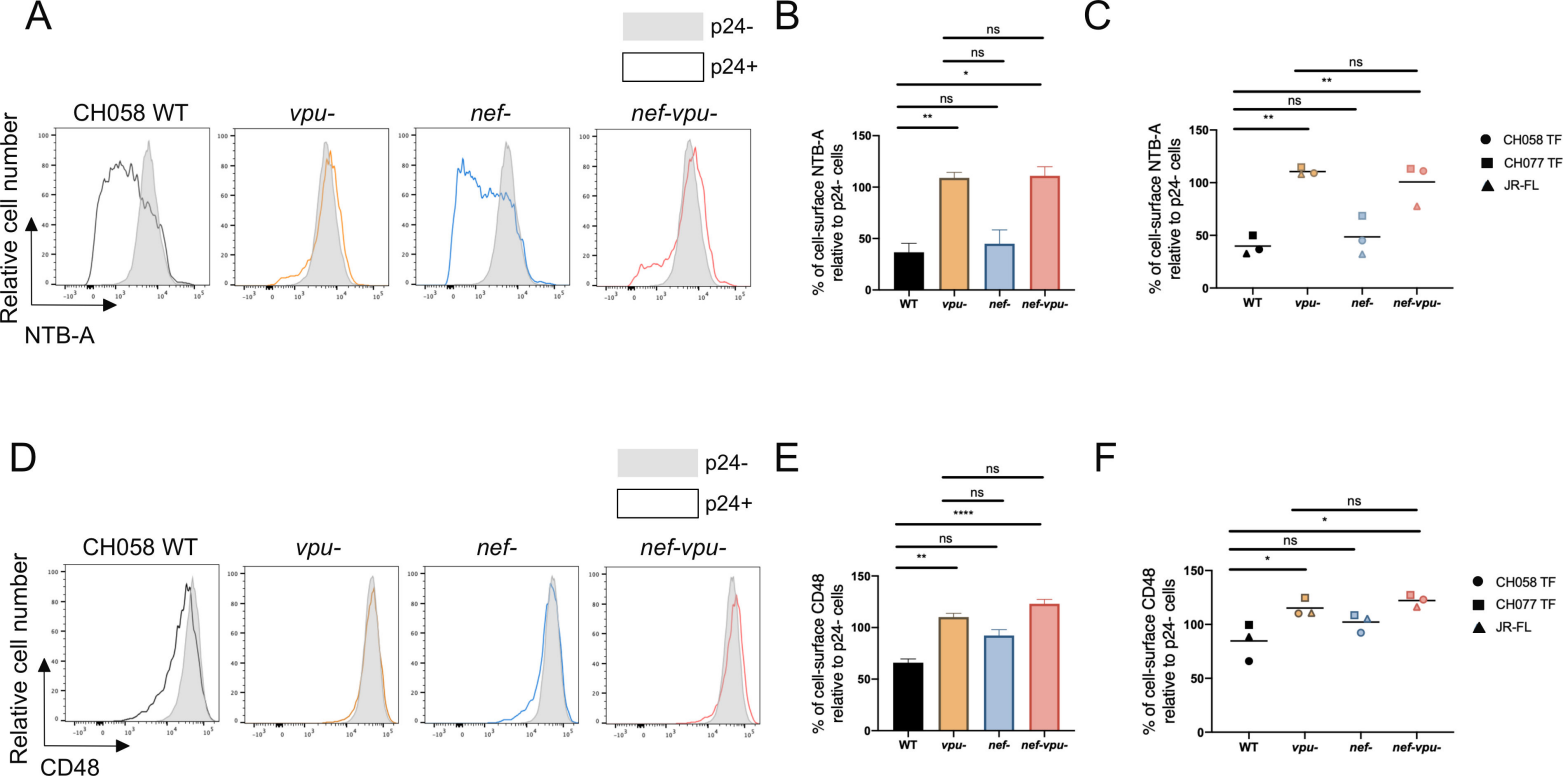
HIV-1 Vpu downregulates CD48. Primary CD4^+^ T cells infected with CH058 TF either WT or defective for Nef, and/or Vpu expression were stained for NTB-A and CD48 cell surface expression 48 h post infection. (**A and D**) Histograms representing cell surface (**A**) NTB-A and (**D**) CD48. (**A and D**) Uninfected bystander p24^−^ cells are shown in gray, while infected p24^+^ cells are shown in black (WT), orange (*vpu*−), blue (*nef*−), or red (*nef-vpu*−). (**B and E**) Bar graphs representing cell surface (**B**) NTB-A or (**E**) CD48 for at least six independent experiments. (**C and F**) Cells were also infected with CH058 TF, CH077 TF, and JR-FL IMC either WT or defective for Nef and/or Vpu expression. Dot plots indicate the mean cell-surface percentage for (**C**) NTB-A and (**F**) CD48 for each virus. Error bars indicate means ± standard errors of the means (SEM). Statistical significance was tested using a (**A–F**) Ordinary one-way ANOVA or Kruskal-Wallis tests based on statistical normality (*P < 0.05; **P < 0.01; ****P < 0.0001; ns, non-significant).

Considering that Vpu is responsible for the downmodulation of NTB-A, we evaluated if this was also the case for CD48. To this end, primary CD4 T cells were infected with the full-length infectious molecular clone (IMC) CH058 TF (WT) or its Vpu-defective counterpart (*vpu*−). Flow cytometry analyses revealed that both NTB-A and CD48 are downregulated in a Vpu-dependent manner (Fig. 2A, B, D, and E; Fig. S1). Since Nef also downregulates multiple ligands of co-activating/activating NK cell receptors (35, 55), sometimes in concert with Vpu (36, 56), its implication in CD48 downregulation was also tested using CH058 TF viruses defective for Nef expression (*Nef− and Nef-Vpu*− viruses). In line with earlier studies (37, 46), the abrogation of Nef expression had no impact on cell-surface levels of NTB-A (Fig. 2A and B; Fig. S1). Likewise, Nef expression did not reduce CD48 levels (Fig. 2D and E and 2; Fig. S1). Similar results were obtained with two other IMCs (CH077 TF and JR-FL) (Fig. 2C and F).

**Fig 3:**
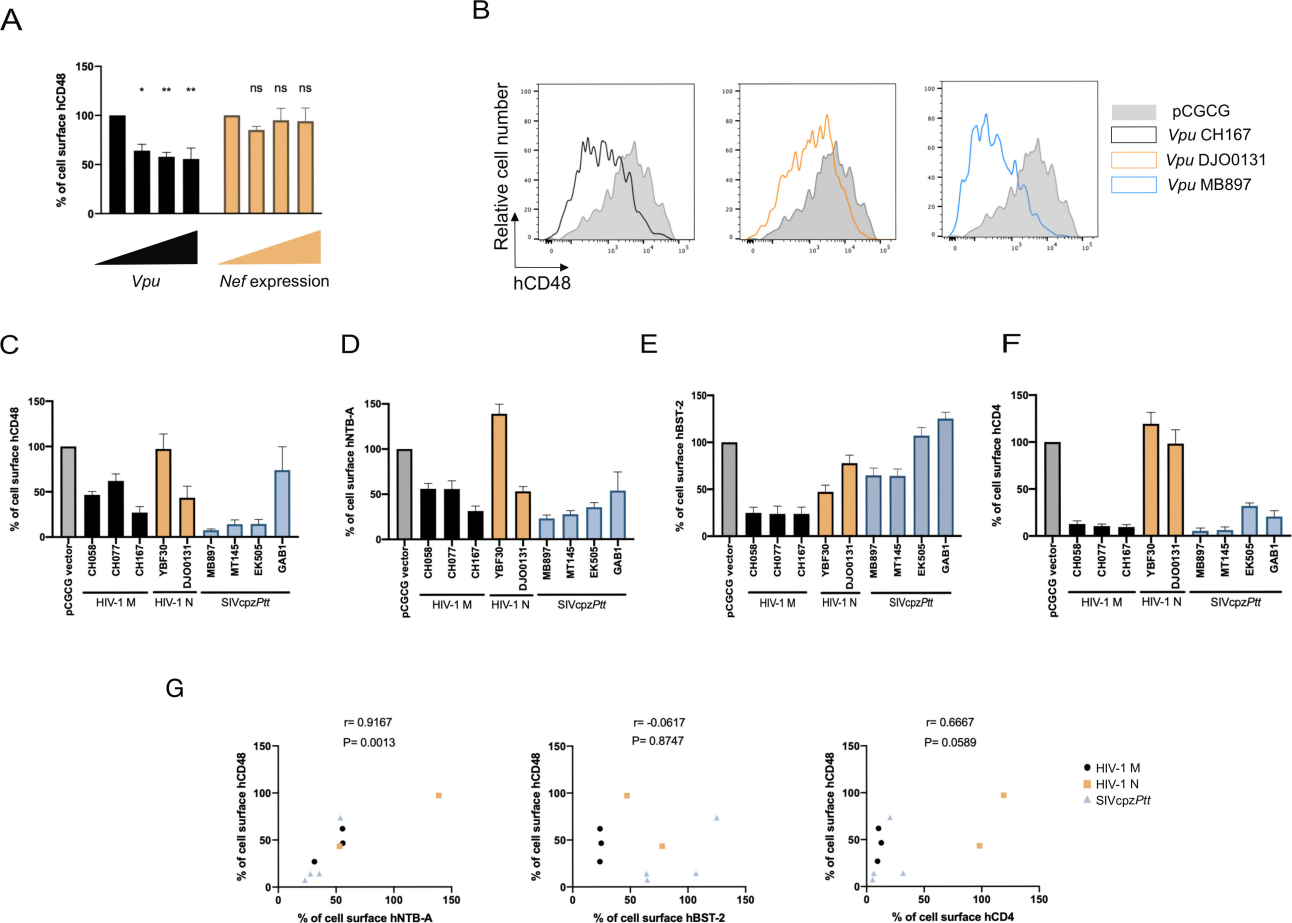
Vpu-mediated downregulation of CD48 is conserved among different primate lentivirus lineages. (**A**) HEK293T cells were co-transfected with a plasmid expressing the hCD48 (0.6 µg) and pCGCG vector expressing eGFP alone or together with CH058 TF *vpu* or *nef* (0, 0.15, 0.3, or 0.6 µg). Cell-surface CD48 was assessed 48 h post transfection and used to calculate the percentage of receptor downregulation. The data represent three independent experiments. (**B–F**) HEK293T cells were co-transfected with plasmids expressing (**B and C**) hCD48 (0.6 µg), (**D**) hNTB-A (1 µg), (**E**) hBST-2 (1 µg), or (**F**) hCD4 (1 µg) and pCGCG vector expressing eGFP alone or together with indicated *Vpu* (0.6 µg). Cell surface levels of NTBA, CD48, BST-2, and CD4 were assessed 48 h post transfection. (**B**) Histograms representing CD48 surface levels on HEK293T cells co-transfected with hCD48 and pCGCG vector expressing eGFP alone or together with indicated *vpu* (**C–F**) Bar graphs indicate the percentage of cell-surface levels of each surface protein detected on cells transfected with Vpu vectors relative to cells transfected with the control vector. Error bars indicate means ± standard errors of the means (SEM). (**G**) Correlation between the percentage of cell-surface CD48 and NTB-A, BST-2, or CD4. Statistical significance was tested using (**A**) one-way ANOVA test and (G) Spearman and Pearson rank correlation test (*P < 0.05; **P < 0.01; ns, non-significant).

To confirm that expression of Vpu alone is sufficient to downregulate cell-surface CD48, we co-transfected HEK293T cells with a plasmid-encoding human CD48 and vectors expressing enhanced green fluorescent protein (eGFP) alone or together with CH058 TF Vpu. Consistent with results generated with infected primary CD4^+^ T cells (Fig. 2), Vpu significantly reduced CD48 cell-surface levels (Fig. 3A). In contrast, no CD48 downregulation was observed upon CH058 TF Nef expression. To determine if this Vpu activity is conserved across lentiviral lineages, we next analyzed a larger panel of Vpu alleles from HIV-1 group M (CH058, CH077, and CH167) and group N (YBF30 and DJO0131) viruses, as well as several SIVcpz*Ptt* strains (MB897, MT145, EK505, and GAB1). Their ability to downregulate cell-surface CD48 and other known Vpu substrates (CD4, NTB-A, and BST-2) was measured by flow cytometry. Group M Vpu proteins downregulated BST-2, CD4, NTB-A, and CD48 to various extents (Fig. 3B–F). As previously reported (57), both HIV-1 N Vpu tested failed to downmodulate CD4 but showed some anti-BST-2 activity. While YBF30 Vpu had no effect on NTB-A and CD48, DJO0131 Vpu exhibited some activity. Strikingly, Vpus from SIVcpz*Ptt* strains also efficiently downregulated cell-surface CD48 as well as CD4 and NTB-A, but not BST-2, consistent with previous studies (57, 58). The capacity to downregulate CD48 and NTB-A was highly correlated (Fig. 3G), suggesting that Vpu proteins have evolved to target both SLAMs. Taken together, these data suggest that Vpu-mediated downregulation of CD48 is a conserved function of the HIV-1/SIVcpz lineage.

### Vpu determinants of CD48 downregulation

**Fig 4:**
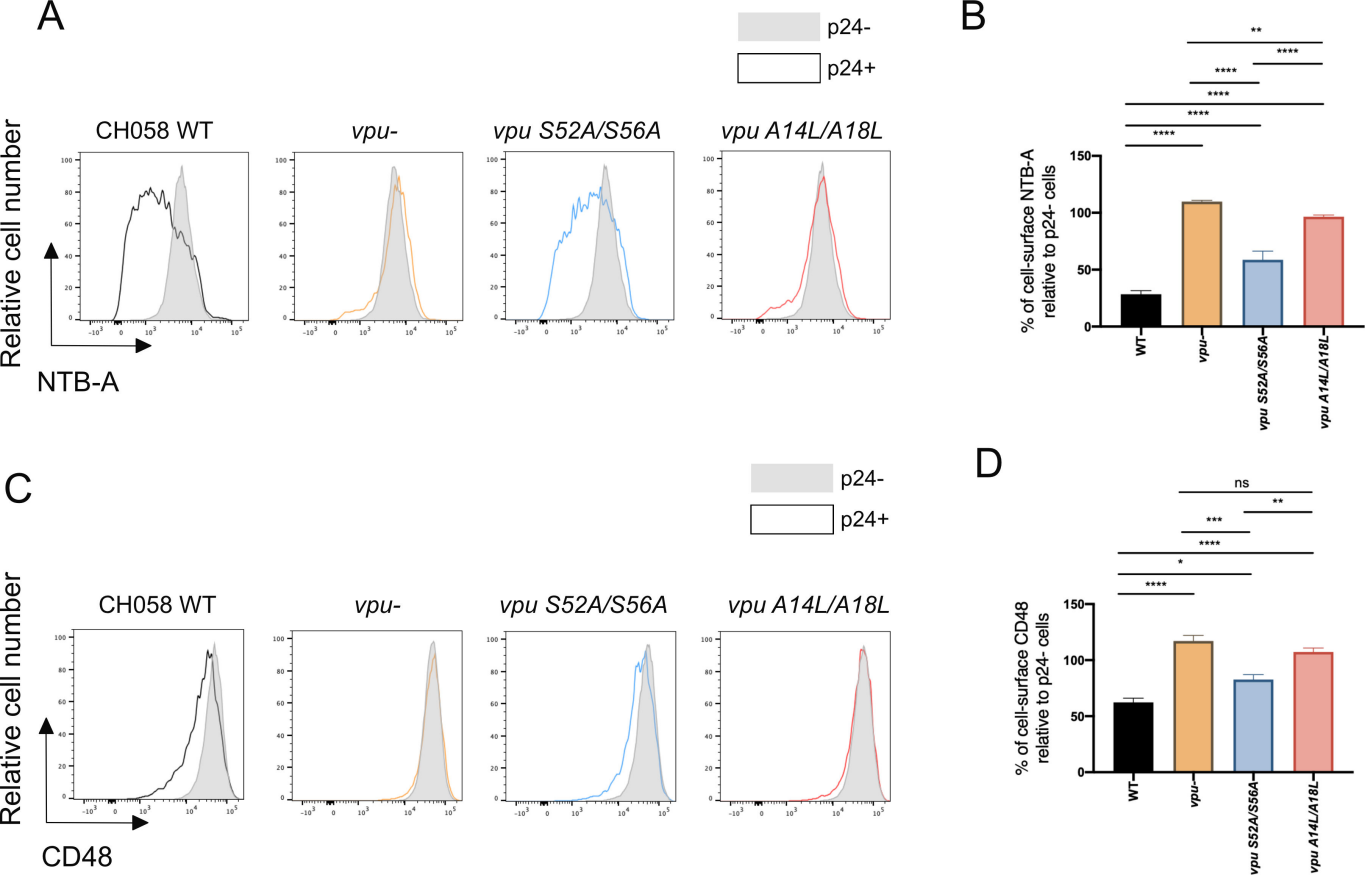
Vpu transmembrane domain and phosphoserine motif are important for CD48 downregulation. Primary CD4^+^ T cells infected with CH058 TF viruses expressing wild-type *vpu* (WT), *vpu S52A/S56A*, *vpu A14L/A18L,* or defective for *vpu* expression (*vpu*−) were stained for NTB-A and CD48 cell-surface expression 48 h post infection. (**A and C**) Histograms representing cell surface (**A**) NTB-A and (**C**) CD48. (**A and C**) Uninfected bystander p24^−^ cells are shown in gray, while infected p24^+^ cells are shown in black (WT), orange (*vpu−*), blue (*vpu S52A/S56A*), or red (*vpu A14L/A18L*). (**B and D**) Bar graph representing the percentage of cell-surface (**B**) NTB-A or (**D**) CD48 for at least five independent experiments. Error bars indicate means ± standard errors of the means (SEM). Statistical significance was tested using (**A–D**) Statistical significance was tested using an ordinary one-way ANOVA (*P < 0.05; **P < 0.01; ***P < 0.001; ****P < 0.0001; ns, non-significant).

We next sought to determine the domains of Vpu responsible for CD48 downmodulation. Given that the transmembrane domain (TMD) of Vpu is required for the downregulation of several transmembrane host proteins, including BST-2, NTB-A, PVR, CD62L, and Tim-3 (37, 46, 59 - 61), we studied its role in CD48 downregulation. Consistent with previous data (46), mutation of the TMD (A14L/A18L) abrogated the capacity of Vpu to downregulate cell-surface NTB-A (Fig. 4A and B; Fig. S2). These same mutations also significantly reduced the capacity of Vpu to downregulate cell-surface CD48 (Fig. 4C and D, Fig. S2). Notably, Vpu TMD mutations restored cell-surface CD48 levels to those obtained upon *Vpu* deletion. The phosphoserine motif of Vpu is important for the recruitment of the SCF^βTrCP^ E3 ubiquitin ligase (62) and, consequently, the capacity of Vpu to downregulate CD4 and BST-2 (63, 64). The introduction of mutations in this dual phosphoserine motif of Vpu (S52A/S56A) also reduced the capacity of Vpu to downregulate both ligands, but the impact of these mutations was much less pronounced compared to the TMD mutations. Altogether, these results implicate the TMD and phosphoserine motif of Vpu in the downregulation of CD48.

### BST-2 upregulation by type I IFN hinders the ability of Vpu to downmodulate CD48

**Fig 5:**
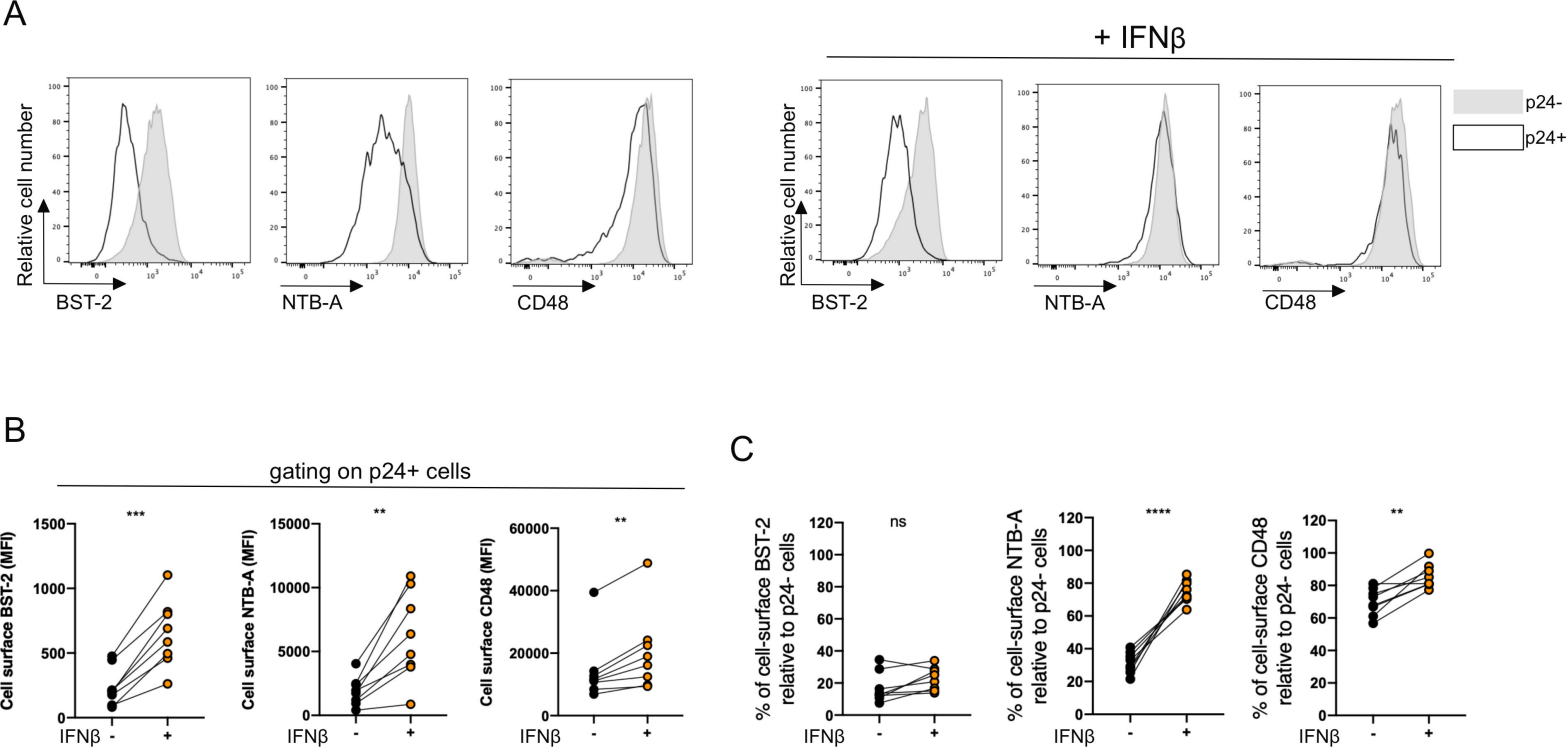
Upregulation of BST-2 by IFN-β treatment affects CD48 downregulation. Primary CD4^+^ T cells infected with CH058 TF WT virus were treated or not with IFN-β 24 h post infection, and cell-surface BST-2, NTB-A, and CD48 levels were assessed 48 h post infection. (**A**) Histograms representing cell surface BST-2, NTB-A, and CD48 levels when (left) untreated or (right) treated with IFN-β. Uninfected bystander p24^−^ cells are shown in gray, while infected p24^+^ cells are depicted in black dot line. (**B and C**) Dots representing (**B**) median fluorescence intensity (MFI) detected on p24^+^ infected cells or (**C**) the percentage of cell-surface BST-2, NTB-A, or CD48 in p24^+^ cells relative to p24^−^ cells in eight independent experiments. Error bars indicate means ± standard errors of the means (SEM). (**B and C**) Statistical significance was tested using paired *t*-tests or Wilcoxon tests based on statistical normality (**P < 0.01; ***P < 0.001; ****P < 0.0001; ns, non-significant).

We previously showed that BST-2 upregulation by type I interferons (IFNs) diminishes Vpu’s polyfunctionality (37). Specifically, the TMD gets occupied by BST-2, reducing the capacity of Vpu to interact with other transmembrane proteins, including NTB-A, PVR, CD62L, and Tim-3 (37, 61). Since the TMD of Vpu is required for CD48 downregulation, we hypothesized that IFN treatment would also alter its capacity to downregulate CD48. We thus infected primary CD4^+^ T cells with CH058 TF; treated the cells with IFN-β (or PBS) 24 h later; and then examined BST-2, NTB-A, and CD48 expression 48 h post infection by flow cytometry (Fig. 5A). As expected, IFN-β treatment increased cell-surface BST-2 levels on p24^+^ cells (Fig. 5B). However, as previously reported (37), BST-2 remained efficiently downmodulated on infected (p24^+^) cells relative to uninfected (p24^−^) cells (Fig. 5C). In contrast, NTB-A and CD48 cell-surface levels were upregulated in infected (p24^+^) cells over uninfected (p24^−^) cells, indicating that downmodulation of both SLAMs was significantly reduced (Fig. 5C). These results suggest that BST-2 upregulation by type I IFN impairs CD48 downmodulation.

### NTB-A and 2B4 similarly trigger NK cell degranulation and ADCC

**Fig 6:**
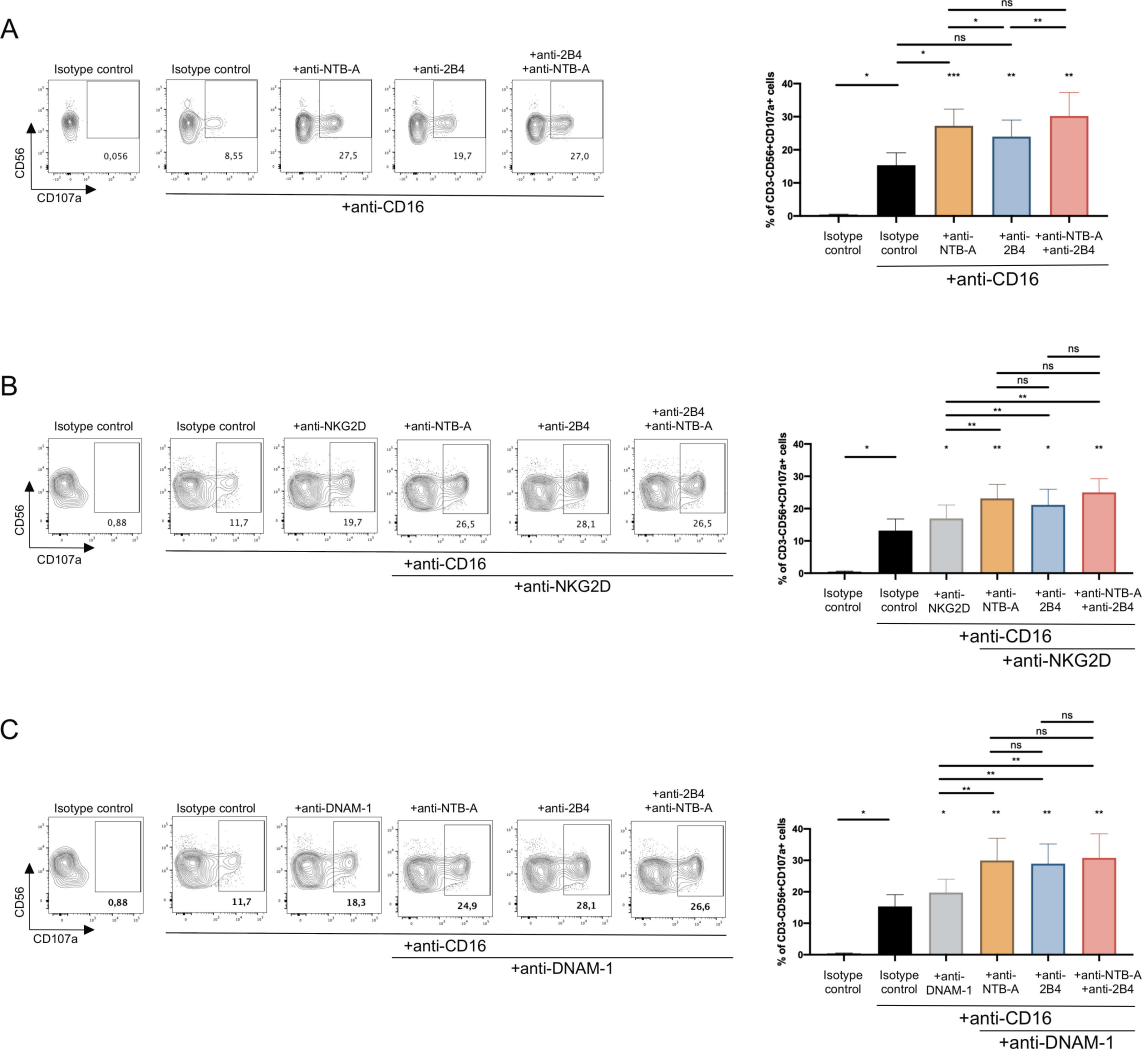
NTB-A and 2B4 trigger NK cell degranulation to a similar extent. P815 cells were incubated with indicated mAbs or a matched IgG isotype. P815 cells were then mixed with purified NK cells and incubated for 4 h. CD3^−^CD56^+^ cells were evaluated for percentage of cell-surface CD107a. Contour plots depict NK cells stimulation. Bar graphs represent the percentage of CD107a expression among CD3^−^CD56^+^ cells. (**A**) Stimulation with P815 cells coated with isotype control Abs or anti-CD16 Abs, +/−anti-NTB-A Abs, and/or anti-2B4 Abs. (**B**) Stimulation with P815 cells coated with isotype control Abs or anti-CD16 Abs, +/−anti-NKG2D, +/−anti-NTB-A Abs, and/or anti-2B4 Abs. (**C**) Stimulation with P815 cells coated with isotype control Abs or anti-CD16 Abs, +/−anti-DNAM-1, +/−anti-NTB-A Abs, and/or anti-2B4 Abs. Statistical differences relative to stimulation with anti-CD16 Abs alone are represented above each antibody tested. Statistical significance was tested using paired *t*-tests or Wilcoxon tests based on statistical normality (*P < 0.05; **P < 0.01; ns, non-significant).

Given that both NTB-A and CD48 are downregulated from the surface of HIV-1 infected cells, we next studied their functional impact on NK cell effector functions using a redirection degranulation assay as previously described (37, 45, 46). Briefly, FcγR^+^ P815 cells were coated with mouse antibodies known to bind NTB-A or 2B4, the receptors that interact with NTB-A and CD48, respectively, or with their matched IgG isotypes. The cells were then incubated with negatively selected human NK cells. In this assay, the Fc portion of the Ab interacts with the FcγR present on the P815 target cells, and the Fab portions bind to receptors on the NK cells, thus inducing NK cell effector functions (Fig. S3). NK cell activation was monitored by the detection of the NK cell degranulation marker CD107a as previously described (37) (Fig. S4). To determine the impact of the two SLAMs on NK cell-mediated ADCC, NK cell stimulation was performed in the presence of a specific Ab engaging the NK cell FcγR CD16. As previously described (37, 45), the engagement of CD16 alone was sufficient to induce NK cell degranulation (Fig. 6A–C). In contrast, stimulation of NK cells via NTB-A or 2B4 alone didn’t trigger NK cells degranulation (Fig. S5). However, stimulation via either NTB-A or 2B4 significantly increased the magnitude of CD16-mediated NK cell degranulation (Fig. 6A), confirming the role of both SLAMs in NK cell-mediated ADCC response (37, 45). Interestingly, stimulation via CD16 as well as both SLAMs did not significantly increase NK cell degranulation compared to stimulation with CD16 and NTB-A (Fig. 6A), suggesting a lack of cooperation between both SLAMs to stimulate CD16-mediated NK cell effector functions. We also compared the capacity of NTB-A and 2B4 to trigger CD16-mediated NK cell degranulation in cooperation with the activating receptors NKG2D and DNAM-1 since their ligands are still present at the surface of HIV-1-infected cells (65 - 69). As previously reported, stimulation of NK cells via NKG2D or DNAM-1 alone did not trigger NK cells degranulation (Fig. S5). However, CD16-mediated NK cell degranulation was increased when stimulating NK cells via NKG2D in agreement with previous results (32). Stimulation via NKG2D and each single SLAM further increased NK cell degranulation compared to NKG2D alone (Fig. 6B). However, stimulation via NKG2D and both SLAMs did not further increase CD16-mediated NK cell degranulation relative to stimulation via NKG2D and a single SLAM (Fig. 6B). Similar results were obtained when stimulating SLAMs together with DNAM-1 (Fig. 6C). Overall, these data indicate that NTB-A and 2B4 can trigger CD16-mediated NK cell effector functions to a similar extent without a functional cooperation.

**Fig 7:**
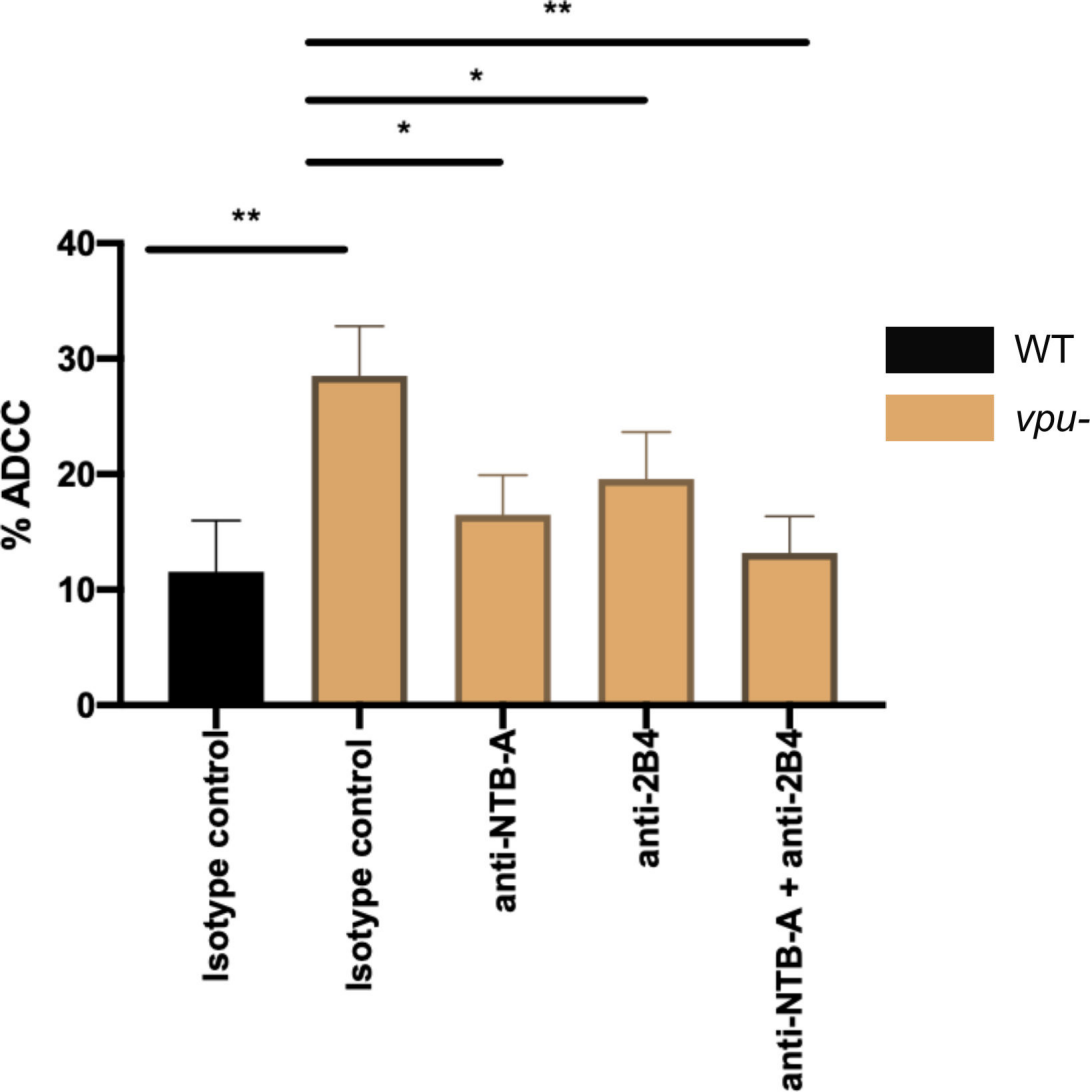
NTB-A and 2B4 engagement enhances ADCC against HIV-1-infected cells. Primary CD4^+^ T cells infected with CH058 TF WT or *vpu*− viruses were used as target cells, while autologous PBMCs were used as effector cells to perform ADCC killing assay. PBMCs were pre-incubated or not with anti-NTB-A and/or anti-2B4 Abs or their matched IgG isotype controls prior to incubation with target cells. Bar graph represents the percentage of ADCC obtained with the bNAb 3BNC117 in seven independent experiments. Statistical significance was tested using one-way ANOVA test (*P < 0.05; **P < 0.01; ns, non-significant).

The potential contribution of Vpu-mediated NTB-A and CD48 downmodulation in protecting HIV-1-infected cells from ADCC was then evaluated using an established FACS-based ADCC assay (18). Briefly, primary CD4^+^ T cells were infected with wild-type (WT) or vpu-deficient (*Vpu*−) CH058 TF IMCs. Forty-eight hours post infection, infected cells were used as targets and autologous peripheral blood mononuclear cells (PBMCs) as effectors. Target cells were incubated with 3BNC117, a broadly neutralizing antibody (bnAb) against the CD4-binding site of HIV-1 envelope glycoprotein known to mediate ADCC (16). We selected 3BNC117 to perform these experiments since it recognizes closed and more open Env conformations (21, 37). The percentage of ADCC was calculated by evaluating the loss of p24^+^-infected cells after incubation with PBMCs in the presence or absence of bnAbs (18). As expected, cells infected with Vpu− virus were more susceptible to ADCC than WT-infected cells (Fig. 7). PBMCs preincubated with blocking antibodies against 2B4 exhibited decreased ADCC activity against these infected cells, implicating the contribution of CD48 in ADCC responses (Fig. 7). Consistent with a similar capacity to trigger NK cell degranulation, blockade of either NTB-A or 2B4 reduced ADCC to the same extent. Blocking both SLAMs only slightly decrease ADCC relative to single SLAM blockade.

## DISCUSSION

Upon engagement by their respective ligands, the SLAM receptors NTB-A and 2B4 sustain and promote NK cell cytotoxicity and cytokine production (65, 70). Given their role in NK cell-mediated antiviral responses, it is not surprising that viruses have evolved different mechanisms to reduce NTB-A and 2B4 triggering. During murine cytomegalovirus (MCMV) infection, the viral protein m154 decreases the cell-surface expression of CD48 to interfere with NK cell cytotoxicity (48). Notably, an MCVM lacking the *m154* gene was found to be attenuated *in vivo,* and viral replication could be restored upon NK cell depletion (48). Certain CMVs and other DNA viruses, including poxviruses and adenoviruses, encode NTB-A and/or CD48 homologs that efficiently interact with their counterparts on NK cells, thereby acting as viral decoy protein (71, 72). For example, a soluble CD48 homolog, A43, encoded by owl monkey CMV, was shown to bind to and mask 2B4, thereby impeding NK cell responses (73). HIV-1 is no exception since this lentivirus has also developed strategies to interfere with SLAM triggering. NTB-A cell-surface downmodulation by HIV-1 Vpu was shown to prevent NK cell degranulation and reduce the susceptibility of infected cells to NK cell responses (37, 46). While previous studies showed that CD48 was downmodulated from HIV-1-infected CD4 T cells *in vitro* (65) and *in vivo* (74), the causes and significance of this observation remained unclear. Here, we provide evidence that the HIV-1 accessory protein Vpu downregulates CD48 from the surface of infected cells and that this serves to evade ADCC responses.

CD48 downmodulation by Vpu was observed with several primary viruses (Fig. 2). While Vpu downregulates a variety of surface molecules in concert with Nef, such as CD4, PVR, and CD62L (36, 37, 56, 75, 76), our results show that Vpu alone is sufficient to downregulate cell-surface CD48 (Fig. 2 and 3). This activity appears to have been maintained throughout evolution. Like for CD4, we show that CD48 is also downregulated by Vpu proteins from SIVcpz*Ptt* strains (Fig. 3). This differs from the ability to downregulate BST-2, which was acquired by HIV-1 Vpu only after the transmission of SIVcpz*Ptt* from central chimpanzees (*Pan troglodytes troglodytes*) to humans. Thus, the ability to downregulate SLAM ligands appears to be a more ancient antiviral function that may also be conserved among the *vpu*-encoding precursors of the HIV-1/SIVcpz lineage. Among the SIVcpz alleles, only GAB1 Vpu failed to efficiently downregulate CD48. Interestingly, this represents the only SIVcpz Vpu allele derived from a tissue culture-adapted strain (77). The other three SIVcpz strains (MB897, MT145, and EK505) were amplified directly from fecal samples of naturally infected chimpanzees (78, 79). The absence of immune pressure during tissue culture amplification could have favored the loss of Vpu functions. Notably, GAB1 Vpu also showed reduced anti-NTB-A activity compared to other tested SIVcpz Vpu alleles. In contrast, Vpu from SIVcpz*Ptt* MB897, the closest relative to HIV-1 group M , appears to have the strongest anti-CD48 activity (80).

CD48 is now part of a growing number of membrane proteins downmodulated by Vpu. As observed for several of them, CD48 downmodulation similarly depends on conserved residues in the TMD of Vpu (Fig. 4). Mutation of these residues restored cell-surface CD48 levels to the levels obtained upon *Vpu* deletion. However, in contrast to other Vpu substrates, CD48 does not have a transmembrane domain but is instead attached to the cell membrane with its C-terminus GPI-anchor (53). As the downmodulation of several host proteins by Vpu requires a physical TMD-TMD interaction (37, 46, 59 - 61), it remains unclear whether Vpu and CD48 interact directly or through an intermediate. Moreover, the fate of CD48 after Vpu engagement remains unclear. Mutation of the Vpu phosphoserine motif had only a minor effect on Vpu-mediated CD48 downmodulation (Fig. 4), suggesting that recruitment of the SCF^βTrCP^ E3 ubiquitin ligase (62) and subsequent degradation, as seen for BST-2 and CD4 (63, 64), is unlikely to be involved. Instead, the downmodulation of CD48 may involve an effect of Vpu on anterograde trafficking, as it has been reported for NTB-A (52) and a potential engagement of the clathrin adaptors AP-1 and AP-2 via its phosphoserine motif (81). Future studies will have to differentiate between these possibilities.

We previously demonstrated that BST-2 upregulation upon type I IFN treatment significantly impaired some Vpu functions, with Vpu preferentially targeting BST-2 over its other targets (37). Specifically, we showed that the occupancy of its TMD by BST-2 compromised its ability to target multiple other partners, notably NTB-A, PVR, CD62L, and Tim-3, that also depended on Vpu TMD interaction (37, 61). Our results here further support this model, as we show that IFN-β-induced BST-2 upregulation reduced the capacity of Vpu to downregulate CD48 from the cell surface (Fig. 4). Acute HIV/SIV infection is characterized by a cytokine storm, which includes high levels of type I IFNs (82 - 84). While BST-2 is upregulated by endogenous type I IFN, studies have demonstrated that counteraction of BST-2 by Vpu confers a selective advantage for viral spread in humanized mice (85 - 87). It is conceivable that BST-2 upregulation during acute infection could therefore affect Vpu polyfunctionality, including its capacity to downregulate CD48 and evade NK cell responses. Further work exploring these possibilities is thus warranted.

**Fig 8:**
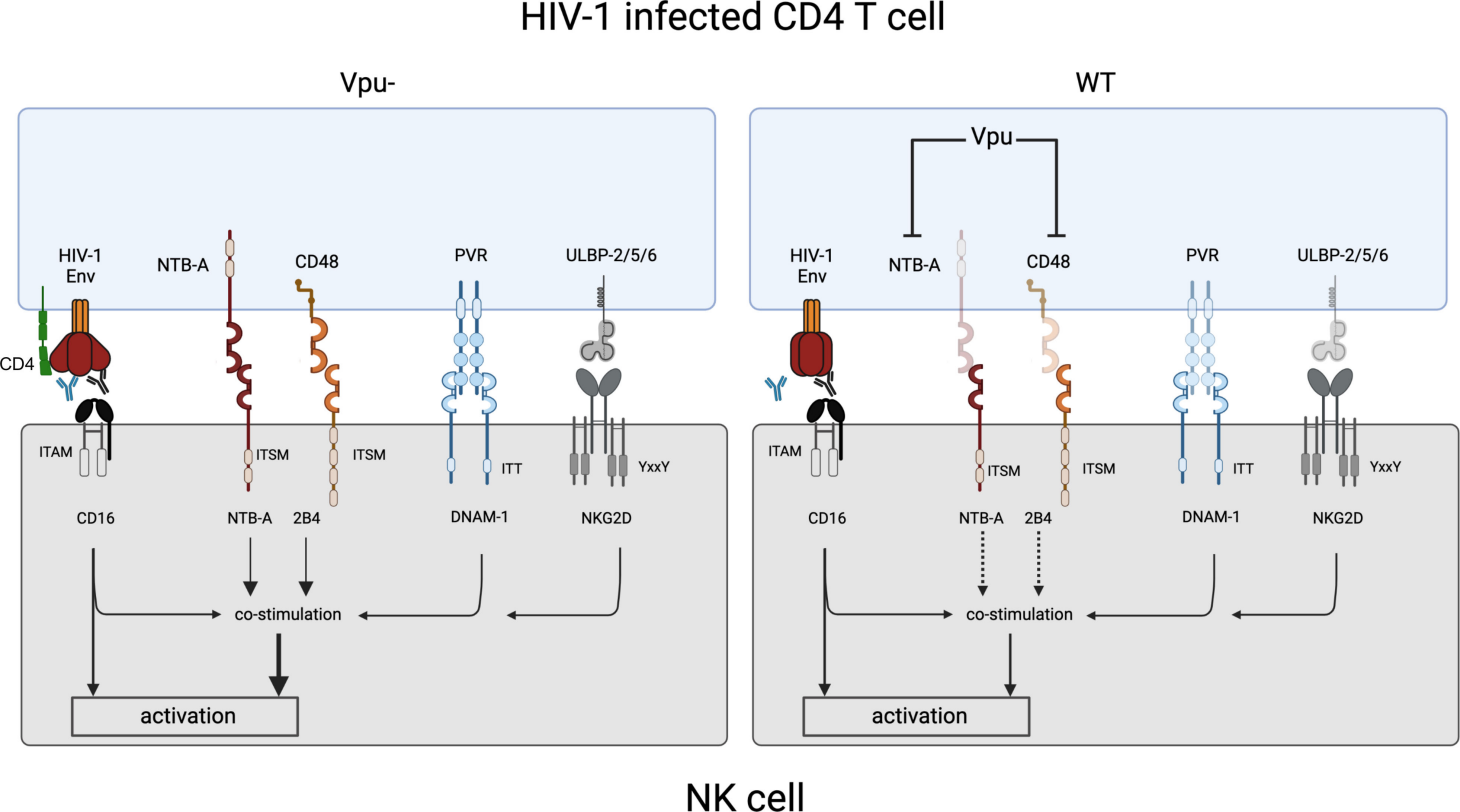
Vpu-mediated downmodulation of NTB-A and CD48 contribute to evade ADCC. CD4 downmodulation by Vpu contributes to evade ADCC mediated by non-neutralizing Abs (depicted in blue) by preventing Env-CD4 interaction. The binding of CD16 to non-neutralizing or broadly neutralizing antibodies (depicted in black) bound to HIV-1 Env can directly activate NK cell effector functions but also in cooperation with others activating and coactivating receptors such as NKG2D, DNAM-1, 2B4, and NTB-A (left panel). The downmodulation of NTB-A and CD48 by Vpu on HIV-1-infected cells contributes to reduce the cooperativity between the SLAMs (NTB-A and 2B4), the activating receptors (DNAM-1 and NKG2D) and CD16 to stimulate NK cell effector functions, thus contributing to evade ADCC (right panel).

We found that 2B4 and NTB-A receptors promote NK cell degranulation and ADCC against HIV-1-infected cells to a similar extent (Fig. 6 and 7). NTB-A and 2B4 have equal potential to induce NK cell degranulation in cooperation with CD16. However, stimulation of NK cells via both SLAMs failed to further enhance NK cell cytotoxicity compared to stimulation with each SLAM alone. Similar results were obtained upon NKG2D or DNAM-1 stimulation (Fig. 6). It has been proposed that synergy among NK cell receptors depends on the utilization of different signaling modules to induce NK cell activation (45, 88). Both NTB-A and 2B4 signaling depend on phosphorylation of their cytoplasmic domains containing several immunoreceptor tyrosine-based switch motifs (ITSM) and the recruitment of the small adapter proteins, including SLAM-associated protein (SAP) and Ewing’s sarcoma-activated transcript-2 (EAT-2), which mediate signal transduction (40, 89 - 91). Similarly, NKp46 and CD16, which share the same signaling pathway, are functionally redundant (45). In contrast, both SLAMs can synergize with the activating receptors NKG2D and DNAM-1 (Fig. 6 and 8) (45), which possess different signaling pathways based on the phosphorylation of their YxxM and ITT-like based motifs, respectively (34, 92).

Consistent with this functional similitude between 2B4 and NTB-A, we found that both receptors have a comparable impact on ADCC responses mediated against HIV-1-infected cells (Fig. 7 and 8). The absence of Vpu expression significantly enhanced ADCC-mediated killing, confirming the protective role of Vpu against this response. 2B4 and NTB-A blockade similarly reduced this response, confirming the role of Vpu-mediated CD48 and NTB-A downmodulation in evading ADCC. The 2B4 receptor has four ITSM-based signals, while the NTB-A protein has only two (40, 93, 94) (Fig. 8). This could explain why CD48 is downregulated less efficiently than NTB-A on target cells but yet has a similar anti-ADCC effect. Consistent with the lack of functional cooperativity between NTB-A and 2B4, blocking both SLAMs only slightly decreased ADCC responses compared to single receptor blockade. This could also be explained by the presence of other NK cell receptors cooperating with CD16 to trigger NK cell effector functions (Fig. 8). Notably, ligands of both DNAM-1 and NKG2D are incompletely downregulated by the virus (65 - 69). Consequently, these receptors have the potential to cooperate with CD16 to trigger NK cell effector functions. Nevertheless, our results support the notion that downmodulation of both SLAM could favor ADCC evasion.

The susceptibility of HIV-1-infected cells to ADCC depends on multiple factors, including the level and the conformation of cell-surface Env, as well as the presence of surface ligands for activating/inhibitory NK cell receptors. Vpu contributes to reduce cell-surface Env level by antagonizing BST-2, a restriction factor tethering viral particles (59, 95). CD4 downregulation by Nef and Vpu also prevents Env-CD4 interaction and the subsequent exposition of CD4-induced Env epitopes targeted by non-neutralizing Abs (12, 13, 96). This was shown to reduce the elimination of HIV-1-infected cells by ADCC mediated by non-neutralizing Abs. To specifically evaluate the impact of Vpu-mediated downmodulation of CD48/NTB-A on ADCC, independently of its impact on CD4, we used here an ADCC-mediating bnAbs that efficiently recognize both closed and more open Env conformations (21). While the impact of Vpu on Env levels/conformation and NK cell activation contribute to evade ADCC, future studies are needed to determine their relative importance.

Taken together, we show that Vpu is responsible for HIV-1-mediated CD48 downmodulation. This activity, which is also conserved in the chimpanzee precursors of HIV-1, contributes to ADCC evasion.

## MATERIALS AND METHODS

### Cell culture and isolation of primary cells

HEK293T human embryonic kidney cells and P815 mouse lymphoblast-like mastocytoma cells (obtained from ATCC) were grown as previously described (12, 69). Primary human peripheral blood mononuclear cells (PBMCs), CD4^+^ T cells, and NK cells were isolated, activated, and cultured as previously described (18, 27). Briefly, PBMCs were obtained by Ficoll density gradient from whole-blood samples obtained from nine different HIV-1-negative donors. CD4^+^ T lymphocytes and NK cells were purified from resting PBMCs by negative selection using immunomagnetic beads per the manufacturer’s instructions (StemCell Technologies, Vancouver, BC, Canada). CD4^+^ T cells were activated with phytohemagglutinin-L (10  µg/mL) for 48  h and then maintained in RPMI 1640 complete medium supplemented with recombinant interleukin-2 (100  U/mL; NIH AIDS Reagent Program). NK cells were isolated and rested overnight in RPMI 1640 complete medium on the day prior to the redirection assays.

### Proviral constructs

Transmitted/Founder (TF) (CH058, CH077) and chronic infectious molecular clones (IMCs) (CH167) were inferred, constructed, and biologically characterized as previously described (18, 97 - 100). The IMC encoding for HIV-1 reference strains JR-FL was described elsewhere (101). CH058, CH077, and JRFL IMCs defective for Vpu and/or Nef expression were previously described (21, 25, 102, 103).

### Expression vectors

Bi-cistronic CMV-based pCGCG vectors expressing the enhanced green fluorescent protein (eGFP) alone or together with Vpu from HIV-1 group M (CH058, CH077, CH167), HIV-1 group N (YBF30 and DJO0131), SIVcpz*Ptt* (MT145, MB897, EK505, and GAB1), or CH058 Nef were previously described (57, 58, 102, 103). Plasmids expressing NTB-A (pQCXIP_human SLAM6/NTB-A) and pcDNA3.1-hCD4 were already described (12, 37). Plasmid expressing hCD48 (pCMV3-CD48) was purchased from Sino Biological (Beijing, China) (HG10797-UT) and Applied Biological Materials (Richmond, BC, Canada) (155490620195). Plasmid expressing hBST-2 (pCMV3-BST-2) was purchased from Sino Biological (Beijing, China) (HG13370-UT).

### Viral production and infections

To achieve a similar level of infection in primary CD4^+^ T cells among the different IMCs tested, VSV-G-pseudotyped HIV-1 viruses were produced and titrated as previously described (104) and detailed in Text S1 in the supplemental material.

### Co-transfection

6 × 10^5^ HEK293T cells were co-transfected with CD48 (0.6 µg), NTB-A (1 µg), BST-2 (1 µg), or CD4 (1 µg) expressor and with bi-cistronic CMV-based pCGCG vectors expressing eGFP alone or together with Nef or Vpu (0.3 or 0.6 µg) using the calcium-phosphate method.

### Antibodies

A detailed list of the Abs used for cell-surface staining and NK cell redirection assay is presented in Text S1 in the supplemental material.

### Type I IFN treatments

IFN-β (Rebif; EMD Serono Inc.) (52) was added to the cells at 1–2 ng/mL at 24 h post infection and 24 h before cell surface staining, as previously described (37).

### Flow cytometry analysis of cell-surface staining

Cell-surface staining of infected primary CD4^+^ T cells was performed 48 h post infection, as previously described (37) and detailed in Text S1 in the supplemental material. The percentage of BST-2, NTB-A, and CD48 levels detected on infected p24^+^ cells relative to uninfected p24^−^ cells was calculated with the following formula: (Median FI detected on p24^+^ cells/Median FI detected on p24^−^ cells) × 100, where Median FI represents the median fluorescence intensity. Alternatively, cell-surface staining was assessed on HEK293T cells co-expressing CD48, NTB-A, BST-2, or CD4 and Nef or Vpu 48 h post transfection. The percentage of BST-2, NTB-A, CD48, and CD4 cell-surface levels detected upon Vpu/Nef expression was calculated with the following formula: (Median FI detected on the surface of cells transfected with Vpu/Nef vector/Median FI detected on the surface of cells transfected with the control vector) × 100, where Median FI represents the median fluorescence intensity. Samples were acquired on an LSRII cytometer (BD Biosciences) or Fortessa A (BD Biosciences), and data analysis was performed using FlowJo v10.5.3 (Tree Star, Ashland, OR, USA).

### Redirection assays

P815 cells were incubated with 5 µg/mL of purified anti-NTB-A Abs, anti-2B4, anti-NKG2D, and/or anti-DNAM-1 Abs or their matched IgG isotype control Abs, in the presence or not of 0.5 µg/mL of anti-CD16 Abs, for 30 min at 4°C. The P815 cells (2 × 10^5^) were then mixed with purified NK cells (1 × 10^5^) and incubated for 4 h at 37°C, 5% CO_2_. After 4 h of coincubation, the cells were stained with fluorochrome-conjugated anti-CD3, CD56, and CD107a Abs. CD3^−^CD56^+^ cells were evaluated for the percentage of cell-surface CD107a expression. Samples were acquired on an LSRII cytometer (BD Biosciences) or Fortessa (BD Biosciences), and data analysis was performed using FlowJo v10.5.3 (Tree Star, Ashland, OR, USA).

### FACS-based ADCC assay

Measurement of ADCC using a FACS-based assay was performed at 48 h post-infection as previously described (18). Briefly, infected primary CD4^+^ T cells were stained with viability dye (AquaVivid; ThermoFisher Scientific, Watham, MA, USA) and cell proliferation dye (eFluor670; eBioscience, San Diego, CA, USA) and used as target cells. Autologous PBMC effectors cells, stained with another cellular marker (cell proliferation dye eFluor450; eBioscience), were added at an effector: target ratio of 10:1 in 96-well V-bottom plates (Corning, Corning, NY, USA). ADCC-mediating mAb 3BNC117 (1 µg/mL) was added to appropriate wells and cells were incubated for 15 min at room temperature. The plates were subsequently centrifuged for 1 min at 300 × *g* and incubated at 37°C, 5% CO_2_ for 5–6 h before being fixed in a 2% PBS-formaldehyde solution. Infected cells were identified by intracellular staining for HIV-1 p24 as described above. Alternatively, effector cells were preincubated for 30 min in the presence of anti-NTB-A and/or anti-2B4 antibodies or their matched IgG isotype control (10 µg/mL) prior to being directly incubated with target cells in the absence or presence of the 3BNC117 bnAbs for blockade experiments. Samples were acquired on a Fortessa cytometer (BD Biosciences), and data analysis was performed using FlowJo v10.5.3 (Tree Star). The percentage of ADCC was calculated by evaluating the loss of p24^+^ infected cells using the following formula: (% of p24^+^ cells in Targets plus Effectors) – (% of p24^+^ cells in Targets plus Effectors plus Abs) / (% of p24^+^ cells in Targets) by gating on infected lived target cells.

### Statistical analysis

Statistics were analyzed using GraphPad Prism version 9.1.0 (GraphPad, San Diego, CA, USA). Every data set was tested for statistical normality, and this information was used to apply the appropriate (parametric or non-parametric) statistical test. *P* values < 0.05 were considered significant; significance values are indicated as *P < 0.05, **P < 0.01, ***P < 0.001, ****P < 0.0001.

## Data Availability

Data and reagents are available upon request.

## SUPPLEMENTAL MATERIAL

Fig. S1 mbio.00789-23-s0001.tif FACS-plots depicting NTB-A and CD48 expression relative to p24 expression.

Fig. S2 mbio.00789-23-s0002.tif FACS-plots depicting NTB-A and CD48 expression relative to p24 expression with Vpu mutants.

Fig. S3 mbio.00789-23-s0003.tif Experimental approach for NK cell redirection degranulation assay.

Fig. S4 mbio.00789-23-s0004.tif NK cell redirection degranulation assay gating strategy.

Fig. S5 mbio.00789-23-s0005.tiff NK cell redirection degranulation assay with single antibody.

Supplemental text mbio.00789-23-s0006.docx Supplemental text and legends.

ASM does not own the copyrights to Supplemental Material that may be linked to, or accessed through, an article. The authors have granted ASM a non-exclusive, world-wide license to publish the Supplemental Material files. Please contact the corresponding author directly for reuse.
