# Supplementary figures and images for: Impact of HIV-1 Vpu-mediated downregulation of CD48 on NK-cell-mediated antibody-dependent cellular cytotoxicity
Source: mBio. 2023 Jul 5;14(4):e00789-23. doi: 10.1128/mbio.00789-23 (PMC10470595; doi:10.1128/mbio.00789-23)

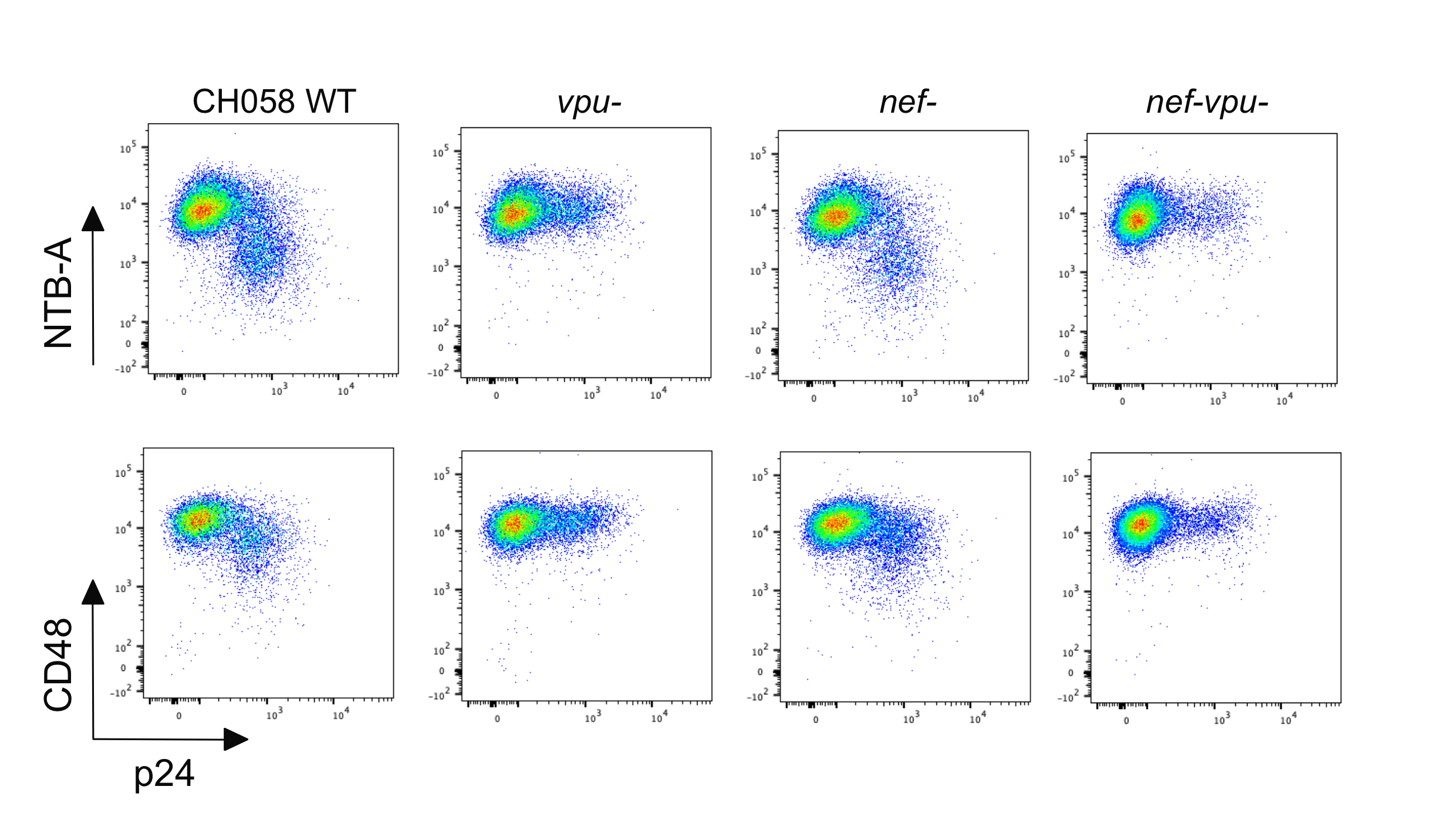

Supplement: Fig. S1 — FACS-plots depicting NTB-A and CD48 expression relative to p24 expression. [file mbio.00789-23-s0001.tif]

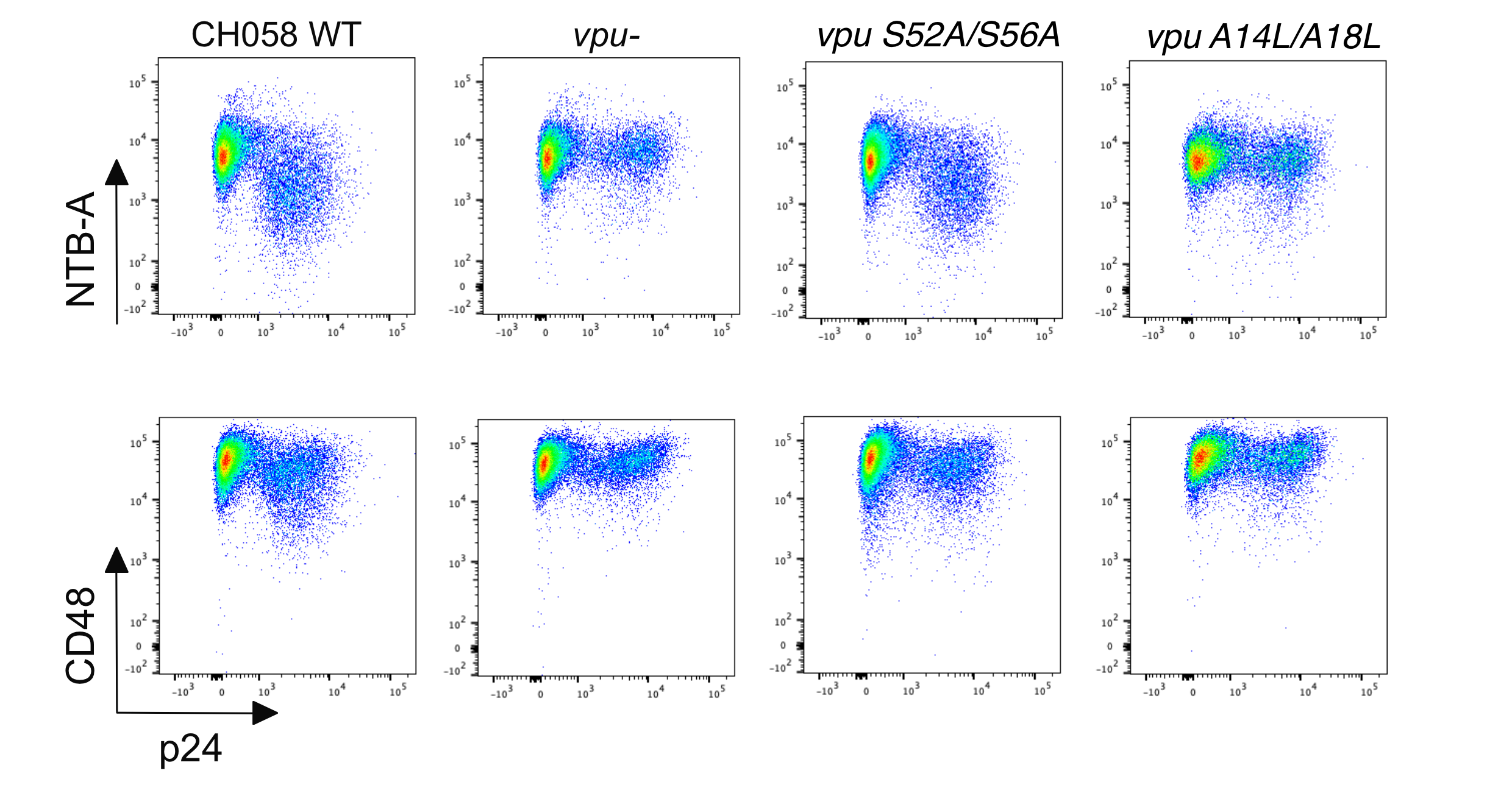

Supplement: Fig. S2 — FACS-plots depicting NTB-A and CD48 expression relative to p24 expression with Vpu mutants. [file mbio.00789-23-s0002.tif]

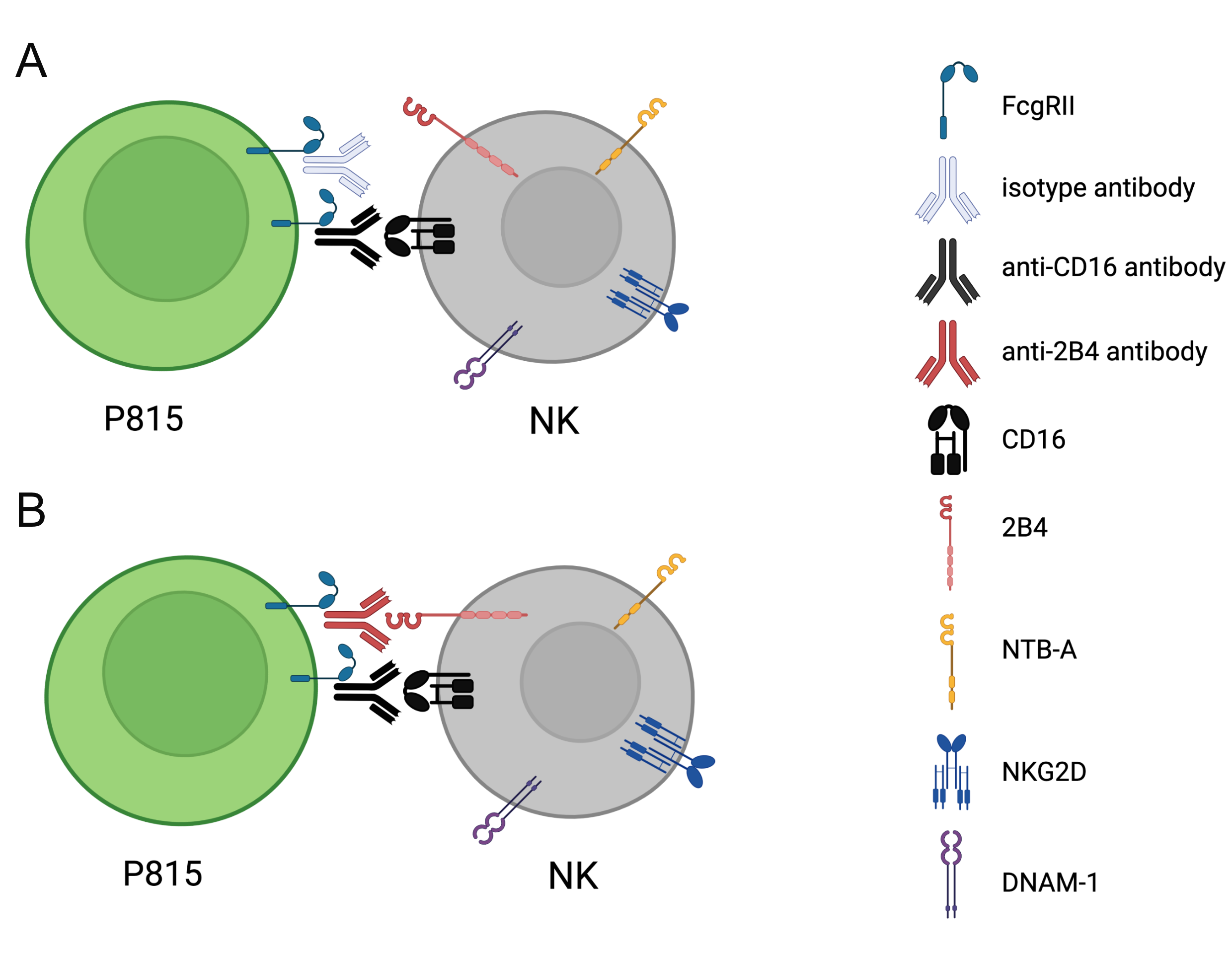

Supplement: Fig. S3 — Experimental approach for NK cell redirection degranulation assay. [file mbio.00789-23-s0003.tif]

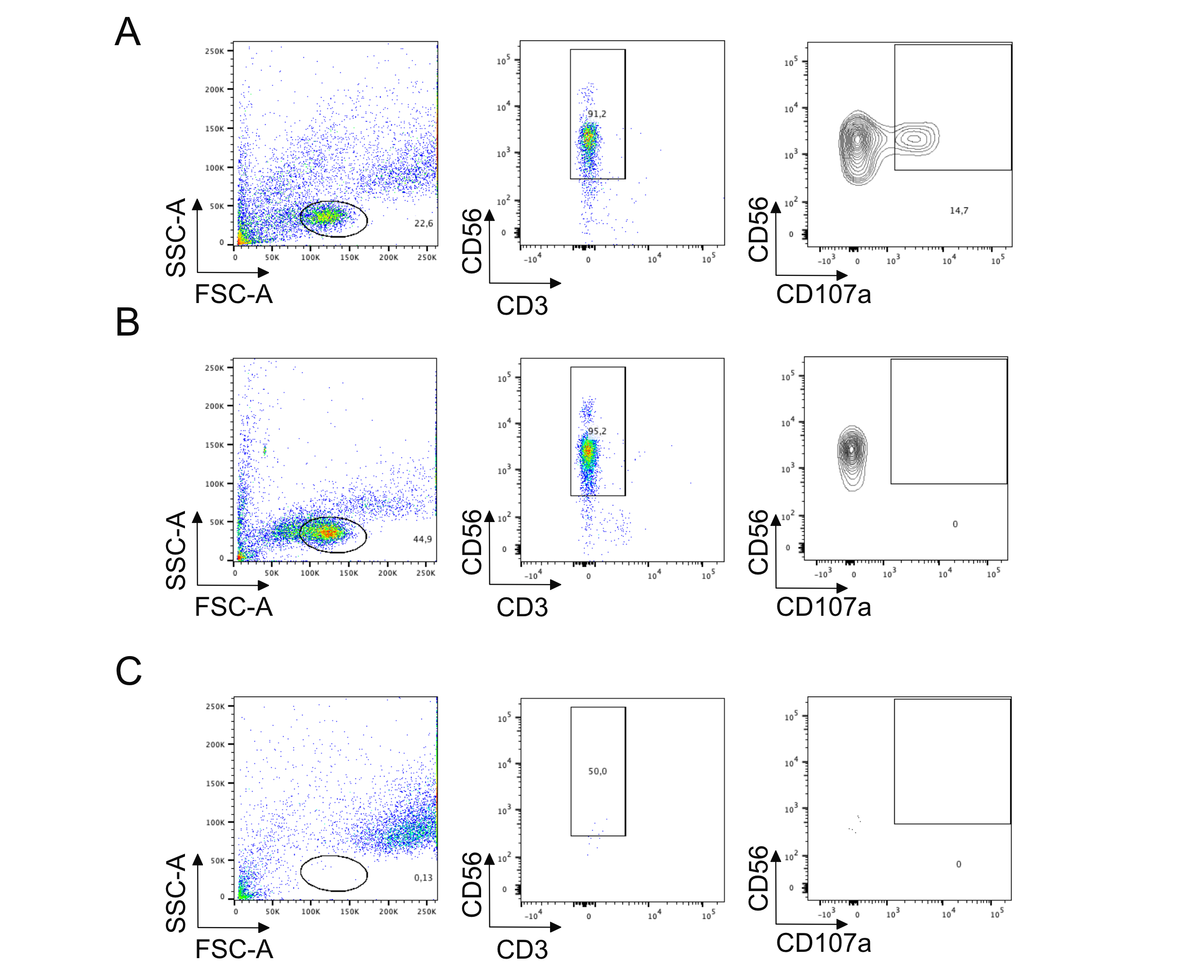

Supplement: Fig. S4 — NK cell redirection degranulation assay gating strategy. [file mbio.00789-23-s0004.tif]

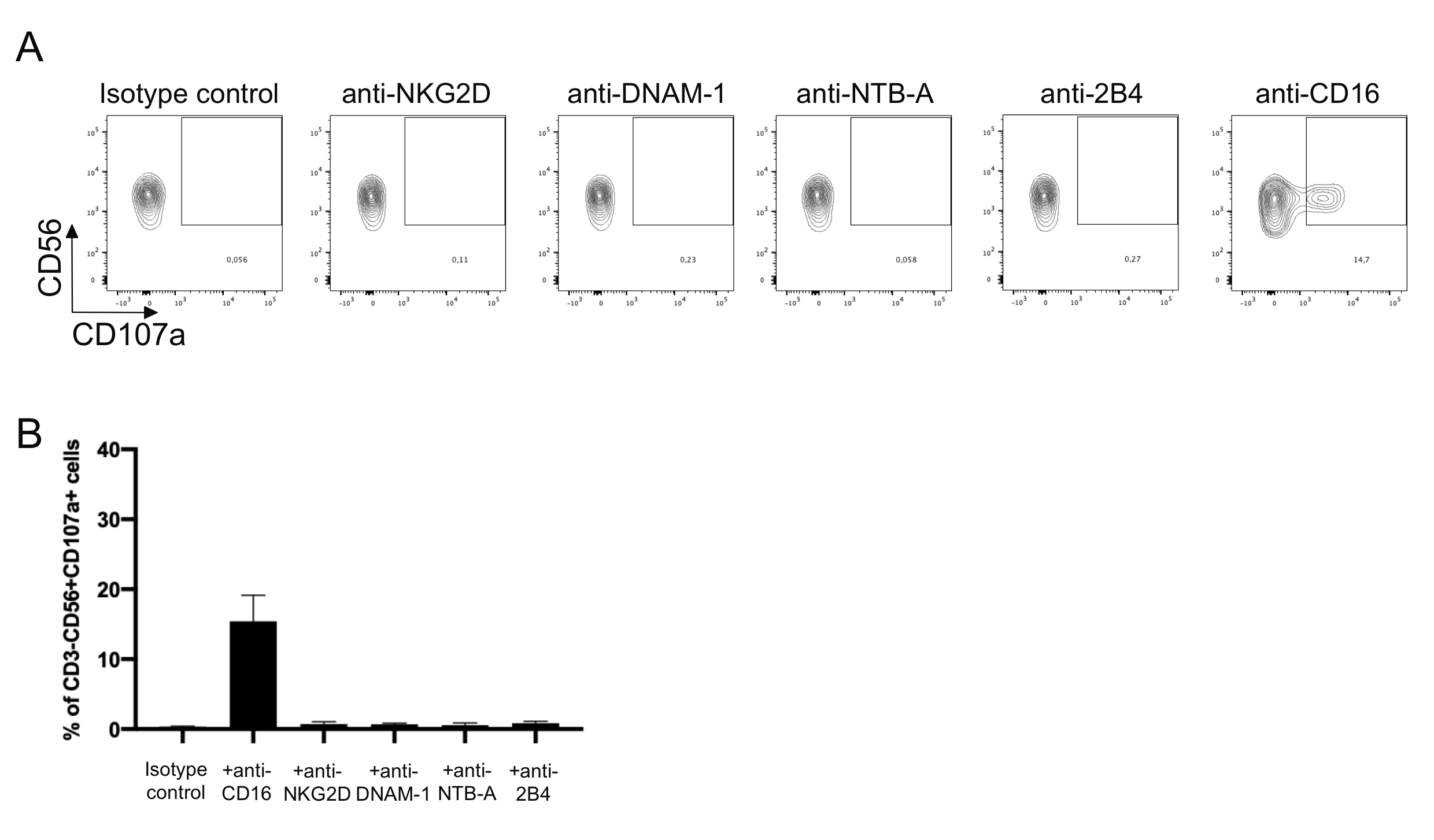

Supplement: Fig. S5 — NK cell redirection degranulation assay with single antibody. [file mbio.00789-23-s0005.tiff]
