# Supplementary material for: Impact of HIV-1 Vpu-mediated downregulation of CD48 on NK-cell-mediated antibody-dependent cellular cytotoxicity
Source: mBio. 2023 Jul 5;14(4):e00789-23. doi: 10.1128/mbio.00789-23 (PMC10470595; doi:10.1128/mbio.00789-23)
Supplement: Supplemental text — and legends. [file mbio.00789-23-s0006.docx]

**SUPPLEMENTAL METHODS**

**Viral production and infections**

Viruses were then used to infect activated primary CD4^+^ T cells from healthy HIV-1 negative donors by spin infection at 800 × *g* for 1 h in 96-well plates at 25 °C. All experiments using VSV-G-pseudotyped HIV-1 isolates were done in a biosafety level 3 laboratory following manipulation protocols accepted by the CRCHUM Biosafety Committee, which respects the requirements of the Public Health Agency of Canada.

**Antibodies**

The following Abs were used as primary Abs for cell-surface staining: mouse anti-human CD352 (NTB-A) (clone NT-7, Biolegend) or Human NTB-A/SLAMF6 APC-conjugated (R&D System), mouse anti-human CD48 (clone BJ40, Biolegend) or APC anti-human CD48 (clone BJ40, Biolegend), rabbit polyclonal anti-human BST2 antiserum (NIH AIDS Reagent Program) or APC anti-human CD317(BST-2, Tetherin) (clone R538E, Biolegend) , mouse anti-CD4 (clone OKT4, eBioscience) or BV-421 anti-CD4 (clone OKT4, Biolegend), and anti-HIV-1 Env mAb 3BNC117 (NIH AIDS Reagent Program). Goat anti-mouse and anti-human antibodies pre-coupled to Alexa Fluor 647 (Invitrogen) were used as secondary antibodies in flow cytometry experiments. The following Abs were used for redirection assays and/or killing blockade experiments: PE-anti-human CD56 (clone NCAM16.2, BD Biosciences), APC-anti-human CD107a (clone H4A3, BD Biosciences), FITC-anti-human CD3 (BD Pharmingen), mouse anti-human CD352 (NTB-A) (clone NT-7, Biolegend), mouse anti-human CD244 (2B4) (clone C1.7, BD Pharmingen), mouse anti-human NKG2D (clone 149810, R&D System), mouse anti-human CD226 (DNAM-1, BD Pharmingen), mouse anti-human CD16 (clone 3G8, Biolegend) and their matched IgG isotype control (clone MOPC-21, Biolegend).

**Flow cytometry analysis of cell-surface staining**

Binding of antibodies to cell-surface NTB-A (5µg/mL), CD48 (5µg/mL), BST-2 (1:100 dilution), CD4 (1:1000 dilution) and anti-HIV-1 Env mAb 3BNC117 (1 µg/mL) was performed at 48h post-infection. Infected cells were stained intracellularly for HIV-1 p24, using the Cytofix/Cytoperm Fixation/ Permeabilization Kit (BD Biosciences, Mississauga, ON, Canada) and the fluorescent anti-p24 mAb (PE-conjugated anti-p24, clone KC57; Beckman Coulter/Immunotech). The percentage of infected cells (p24^+^) was determined by gating the living cell population using a viability dye staining (Aqua Vivid, Thermo Fisher Scientific).

**Supplemental Figure legend**

**Figure S1. FACS-plots depicting NTB-A and CD48 expression relative to p24 expression.** Primary CD4^+^ T cells infected with CH058 TF either WT or defective for Nef, and/or Vpu expression were stained for NTB-A and CD48 cell surface expression 48h post-infection.

**Figure S2. FACS-plots depicting NTB-A and CD48 expression relative to p24 expression with Vpu mutants**

Primary CD4^+^ T cells infected with CH058 TF viruses expressing wild-type *Vpu* (WT), *Vpu S52A/S56A*, *Vpu A14L/A18L* or defective for *Vpu* expression (*Vpu-*) were stained for NTB-A and CD48 cell surface expression 48h post-infection.

**Figure S3. Experimental approach for NK cell redirection degranulation assay.**

P815 mouse lymphoblast-like mastocytoma cells expressing FcγRII are coated with mouse anti-human antibodies against hCD16, hNKG2D, hNTB-A or h2B4. Matched IgG isotype control antibody was also added to maintain the concentration of antibodies between conditions. P815 cells are than mixed with purified NK cells for 4 hours. (A) Scheme depicting the NK cells redirection degranulation assay with a single antibody. Combination of P815 cells coated with isotype control and anti-CD16 Abs interacting with CD16 on NK cells. (B) Scheme depicting the NK cells redirection degranulation assay with multiple antibodies. Combination of P815 cells coated with anti-CD16 and anti-2B4, interacting with CD16 and 2B4 on NK cells.

**Figure S4. NK cell redirection degranulation assay gating strategy.**

NK cells were gated based on FCS-A/SSC-A parameters. NK cells population was than selected based on CD56^+^/CD3^-^ expression. CD56^+^/CD3^-^ cells were than assessed for CD107a expression. Shown are examples of gating strategy for (A) NK cells incubated with P815 coated with anti-CD16 Abs, (B) NK cells alone or (C) P815 alone.

**Figure S5. NK cell redirection degranulation assay with single antibody.** P815 cells were incubated with indicated mAbs or a matched IgG isotype. P815 cells were then mixed with purified NK cells and incubated for 4 hours. CD3^-^/CD56^+^ cells were evaluated for percentage of cell-surface CD107a. (A) Contour plots depict NK cells stimulation when P815 cells were coated with isotype control Abs or anti-NKG2D, anti-DNAM-1, anti-NTB-A, anti-2B4 or anti-CD16. (B) Bar graphs represent the percentage of CD107a expression among CD3-CD56+CD107a+ in at least 3 independent experiments.
